# Does *sod1* encode a molecular clock? Mutations that mimic asparagine deamidation inhibit heterodimerization with ALS-mutant SOD1

**DOI:** 10.1039/d5cb00225g

**Published:** 2026-01-23

**Authors:** Mayte Gonzalez, Travis J. Lato, Emily A. Alonzo, Soeun Park, Morgan T. Green, Natalia Soto-Rodriguez, Bryan F. Shaw

**Affiliations:** a Department of Chemistry and Biochemistry, Baylor University Waco TX USA bryan_shaw@baylor.edu

## Abstract

The self-exchange of subunits by protein homodimers is a common protein–protein interaction *in vivo.* In heterozygous genetic disorders involving homodimeric gene products, both mutant and WT proteins can exchange subunits (heterodimerize). This form of heterodimerization can be analytically challenging to study. In this paper, we used capillary electrophoresis to investigate how deamidation of multiple asparagine residues (to aspartate) in homodimeric Cu, Zn superoxide dismutase-1 (SOD1) affected the rate and free energy of heterodimerization between WT and mutant SOD1 that cause amyotrophic lateral sclerosis (ALS). To model asparagine deamidation, Asn to Asp substitutions were introduced at five Asn residues predicted to undergo the most rapid deamidation in SOD1 (N26D, N131D, N139D, N65D, N19D). This model of penta-deamidated SOD1 did not heterodimerize with WT SOD1 or E100K SOD1 (linked to ALS). In contrast, the quad-variant N26D/N131D/N139D/N19D SOD1 did heterodimerize. These results suggest that the WT SOD1 protein has an intrinsic “timer” or “molecular clock” (as spontaneous Asn deamidation has been described) that effectively stops its heterodimerization after the SOD1 protein has existed in solution for ∼3 months.

## Abstract 2

The self-exchange of subunits by protein homodimers is a common protein–protein interaction *in vivo.*

## Graphical abstract

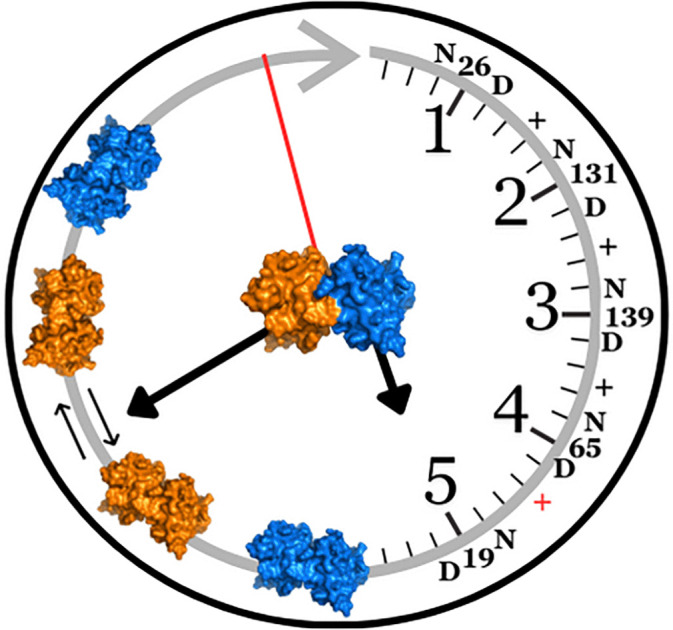

## Introduction

The homodimerization of folded proteins is one of the most common types of protein–protein interactions.^1–4^ The natural occurrence of homodimers is predicted to be widespread *in vivo*, as roughly 25% of proteins exist as dimers when crystallized.^5–7^ The exact biological functions of dimerization are not completely understood but can involve allosteric regulation, ligand binding, protection against misfolding and control of proteolysis.^8–14^

The homodimeric metalloprotein superoxide dismutase-1 (SOD1) is causally linked to amyotrophic lateral sclerosis (ALS)^15^ and might play some role in Parkinson's disease.^16,17^ In cases of familial ALS (fALS) linked to SOD1, the exchange of subunits between ALS-mutant and wild-type (WT) SOD1 homodimers might be important in triggering the neurotoxicity of SOD1.^18–22^ For example, the co-expression of WT SOD1 appears to enhance the toxicity of mutant SOD1 in fALS.^23,24^ The mechanism of this synergy remains unknown, however it is possible that WT SOD1 somehow promotes mutant toxicity *via* heterodimerization.^24–26^

**Fig. 1:**
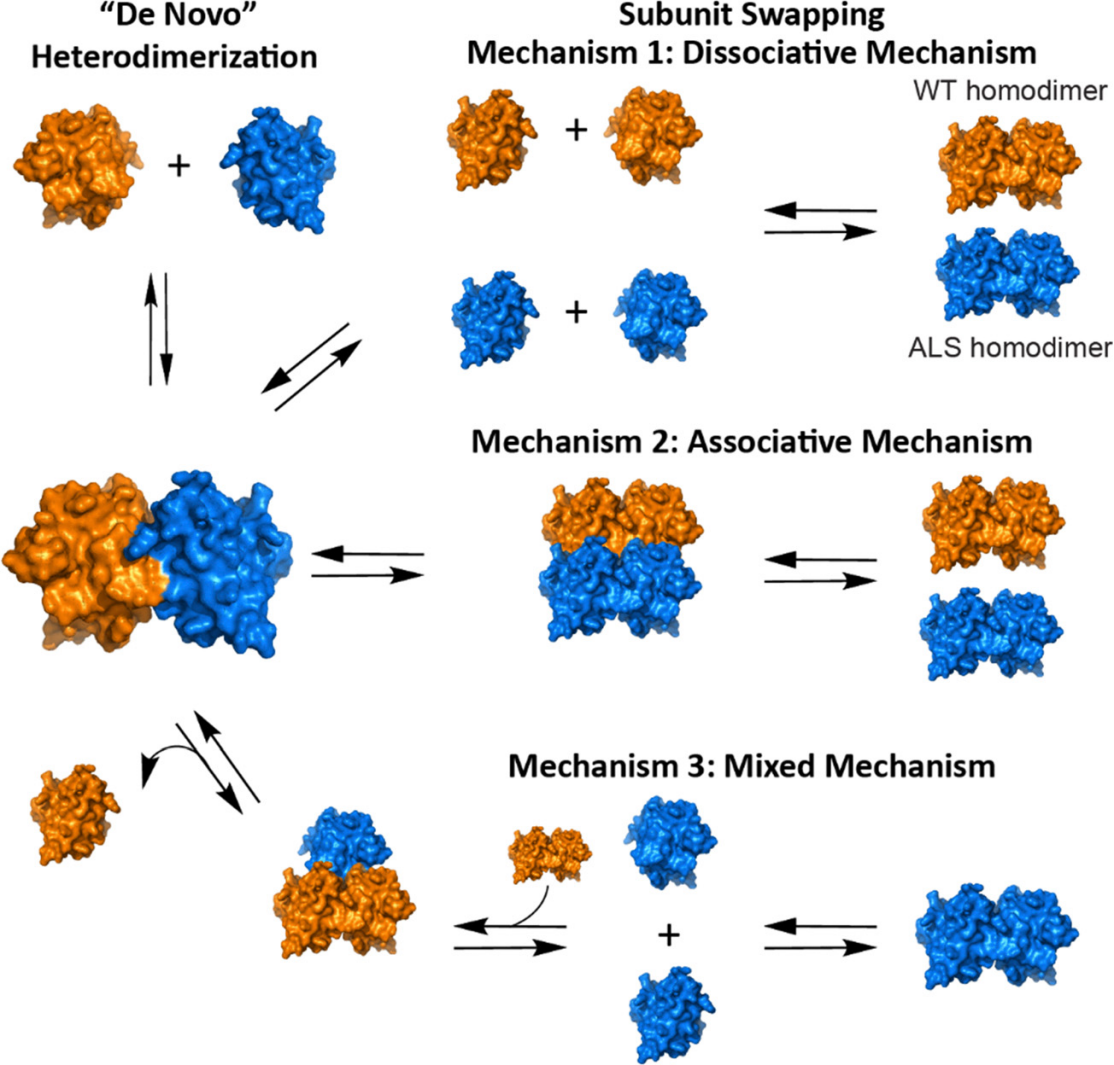
Four possible mechanisms of heterodimerization of WT SOD1 and mutant SOD1.

With regards to heterodimers of any mutant and WT protein—including SOD1—there are two types of heterodimerization processes: (i) *de novo* heterodimerization and (ii) subunit swapping (Fig. 1).^27^*De novo* heterodimerization involves the formation of heterodimers from nascent monomers of WT and mutant protein (that were not previously dimerized).^25^ The second type (subunit swapping) involves the exchange of subunits between existing homodimers *via* dissociative, associative, or mixed mechanisms (Fig. 1).^18,27^

In the case of WT and ALS-mutant SOD1, heterodimerization (subunit swapping) can be thermodynamically favorable (slightly), with Δ*G*_Het_ values ranging from −3.06 ± 0.12 kJ mol^−1^ to −1.21 ± 0.31 kJ mol^−1^ depending upon the mutant and metalation state.^18^ The rates of subunit swapping are within the lifetime of SOD1 in motor neurons and other cells, with half-lives (*t*_1/2_) of heterodimerization ranging from *t*_1/2_ 0.3 to 13 h.^18,28^ Heterodimerization can be promoted by oxidative damage to SOD1.^28^ For example, the oxidation of Cys111 in WT SOD1 promotes its heterodimerization with mutant SOD1.^28^ Oxidation of Cys111 increased rates of heterodimerization ∼3 fold and lowered Δ*G*_Het_ by ∼ΔΔ*G*_Het_ = −5.11 ± 0.36 kJ mol^−1^.^28^ The effects of other post-translational modifications (*e.g.*, histidine and tryptophan oxidation,^29,30^ phosphorylation,^31^ palmitoylation,^32^ and deamidation^33^) on heterodimerization have not been studied.

**Fig. 2:**
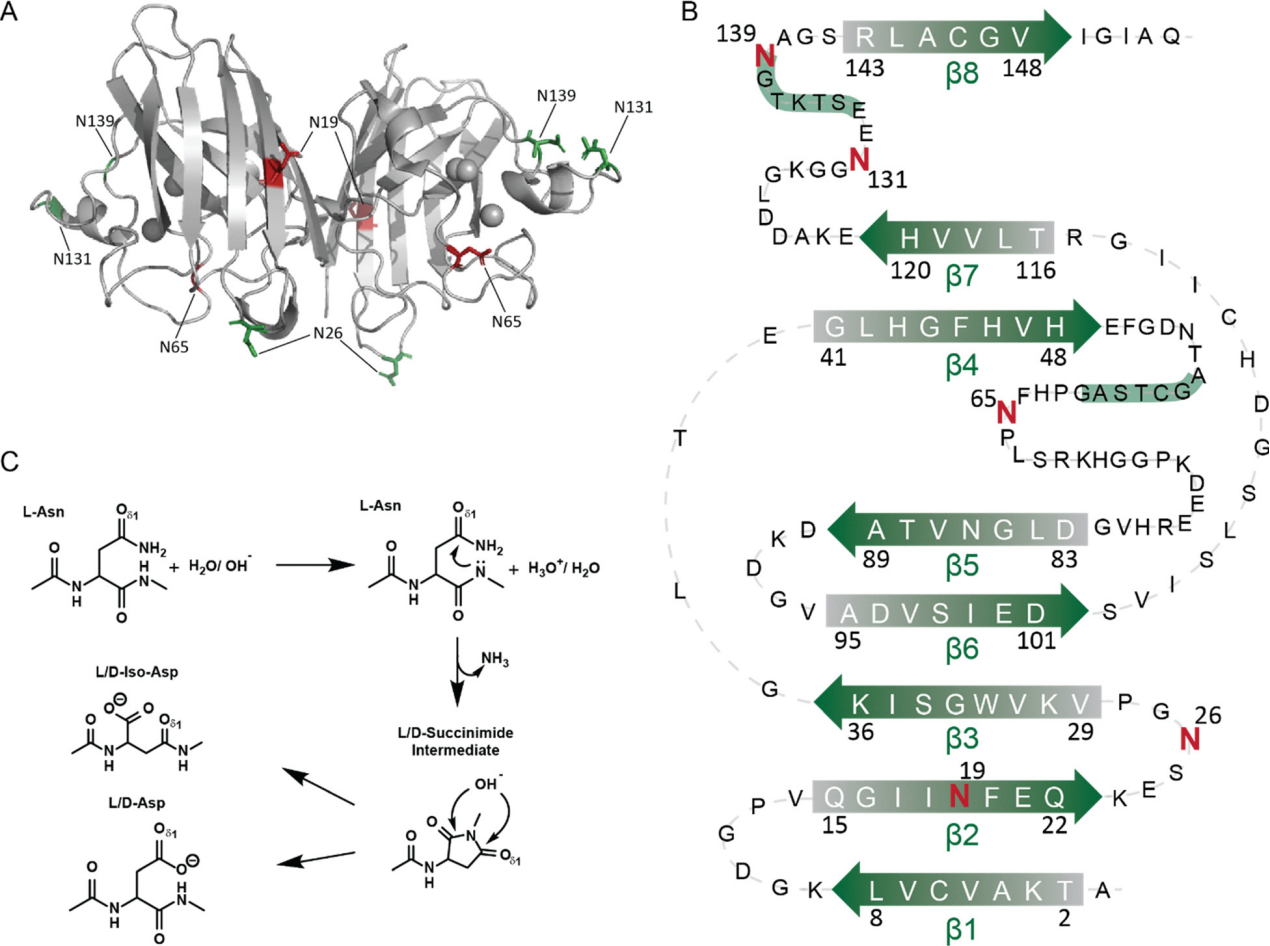
(A) Homodimeric structure of WT SOD1 (PDB:2C9V) showing the asparagine sites in green (N26, N131, N139) and red (N65, N19). (B) A secondary structure map of WT SOD1 shows the location of each asparagine. (C) Reaction mechanism of spontaneous (non-enzymatic) deamidation of asparagine to aspartic acid.

Here, we focus on asparagine deamidation—the non-enzymatic, slow conversion of Asn to Asp—often described as a “molecular clock”.^34–36^ SOD1 is susceptible to deamidation at multiple sites because of its primary structure (including XNG sequences), intrinsically disordered loops, solvent-exposed Asn residues, and long neuronal lifetime (Fig. 2).^37,38^ For example, N26 in human SOD1 is solvent-exposed and flanked by G27, making it prone to rapid deamidation (theoretical half-life ∼71 days).^39^ The Robinson algorithm predicts that the seven Asn residues in SOD1 will deamidate in the order: N26 > N131 > N139 > N65 > N19 > N53 > N86.^33,39^ Consistent with this, approximately 20% of N26 in SOD1 is deamidated, when SOD1 is isolated from human erythrocytes.^33^ Notably, the deamidation of three Asn residues in SOD1 (N26, N131, and N53) were reported in SOD1 proteins isolated from the spinal cord of ALS patients (post-mortem).^40^

In this paper, we are primarily interested in determining how successive deamidation reactions of multiple Asn (to Asp) affect the rate and Δ*G*_Het_ of heterodimerization between unmodified WT or ALS-mutant SOD1. We model asparagine deamidation with Asn/Asp substitutions because Asn/Asp substitutions are chemically equivalent to products of Asn deamidation to Asp (excluding iso-aspartate products). We generated SOD1 variants containing four or five deamidations, referred to as quad-deamidated (QD) and penta-deamidated (PD), respectively. While it is possible that one Asn residue that is predicted to deamidate slowly or “last” (*e.g.*, N53 or 86) might deamidate more rapidly than expected or out of sequence, the present study sought to first examine the effects of deamidation on Asn residues in the sequential order predicted by Robinson's algorithm. This plan is based on the rationale that it is less probable to find N65 or N53 deamidated without also having *at least* the extremely fast N26 and N131 deamidated. Even if the SOD1 protein were to misfold (or unfold) to expose N53 or N65 for deamidation, there are still primary sequence determinants that cannot be ignored (*e.g.*, N65 has a proline on its C-terminal flank that diminishes the intrinsic rate of deamidation). We detect heterodimerization with capillary zone electrophoresis (CE). Capillary electrophoresis is one of the few tools that can rapidly measure the heterodimerization of two proteins that only differ by a single amino acid residue and, in the case of Asn/Asp substitutions, a single oxygen and one or two hydrogen atoms.^18,28,41^

## Materials and methods

### Purification and demetallation of SOD1

Purification of recombinant SOD1 (WT and mutants) were expressed in *S. cerevisiae*, following previously published methods.^42^ EG118Δsod1 yeast was used for the transfection of Yep351-hSOD1 plasmids. Primary cultures were then grown from the yeast stocks to OD_600 nm_ ∼ 1.5 (approximately 36 hours) in YPD media and then transferred into large secondary cultures for ∼7 days. Grown cells were then lysed and purified through ammonium sulfate precipitation and three chromatographic separations: hydrophobic interaction chromatography, ion exchange chromatography, and size exclusion chromatography. SDS-PAGE was used for characterization of SOD1 solution, and protein concentrations were determined *via* UV/vis spectroscopy *ε*_280 nm_ = 10 800 cm^−1^ M^−1^.

### Trypsinization and mass spectrometric analysis of SOD1

Each SOD1 mutant analog was confirmed *via* trypsin digest with tandem mass spectrometry. All protein solutions were incubated with 2 mM dithiothreitol (DTT) and 1 : 20 trypsin (Gold Promega®, WI, USA) and was incubated overnight at 37 °C. All experiments were performed in 25 mM Tris-gly (pH 8.3). Following digestion, each sample was injected into the LTQ LX/Orbitrap Discovery LC/MS instrument (Thermo Scientific™) to acquire mass spectra for peptides.

### Electrospray ionization mass spectrometry

WT, PD, and QD SOD1 were prepared by diluting with 0.2% formic acid and removing salt prior to running on an electrospray ionization-mass spectrometry (ESI-MS) using a ThermoFisher ™ Discovery Orbitrap.

### Demetallation of SOD1

Sequential dialysis was used for demetallation of all purified SOD1 solutions by a series of three buffers: (i) 100 mM sodium acetate, 5 mM EDTA, pH 3.8; (ii) 100 mM sodium acetate, 100 mM NaCl, pH 3.8; and (iii) 100 mM sodium acetate, pH 5.5, over 6 days. All glassware and dialysis materials were rinsed with ultra-pure Milli-Q water followed by 10 mM EDTA. Inductively coupled plasma-mass spectrometry (7900 ICP-MS, Agilent Technologies) was used to verify full demetallation of SOD1. Singly charged atomic ions (M^+^) were collected. The dwell time was 1 ms and the collision gas used for ICP-MS was helium. Purified protein was confirmed to be metal-free if containing <0.1 equivalents of metal were present per dimeric SOD1.

### Capillary electrophoresis (CE)

As previously described, heterodimerization were initiated by mixing 15 µM of each homodimer for an equal concentration of 30 µM total SOD1.^18,28^ All capillary electrophoresis experiments were performed on a Beckman P/ACE instrument in a bare fused silica capillary. All experiments were performed at 29 kV at 22 °C and maintained at this temperature by a liquid cooled outer jacket. The capillary was reconditioned between each injection run with 0.1 M KOH, Milli-Q water, and 10 mM potassium phosphate at pH 7.4. 100 mM dimethylformamide (DMF) was used in protein samples as a neutral marker to measure electroosmotic flow.

### Homodimer validation for penta deamidated

Solution sample containing myoglobin, bovine SOD1, and PD SOD1 were separated through size exclusion chromatography (SEC) with Sephadex G-75 gel filtration media, followed by SDS-PAGE and mass spectrometry for protein content separation. The column was run at room temperature using 20 mM phosphate buffer, 100 mM sodium chloride.

### Remetallation of SOD1 with zinc sulfate

All SOD1 protein was metalated by titrating four equivalents of ZnSO_4_ per dimer. The protein solution was confirmed to be 4Zn-SOD1 (fully metalated) *via* ICP-MS. Protein was considered fully metalated if the protein solution contained >3.7 equivalents of zinc and <0.05 equivalents of zinc of buffer per dimer.

### Measurement of heterodimerization thermodynamics

Heterodimerization reactions were monitored by CE over 48 h, until equilibrium was reached. All electropherograms were processed in OriginPro software using the skim baseline-skimming peak integration as previously described.^18,27,28^ Each heterodimerization experiment was run in triplicate, with 36 replicates in each set. Integrated areas for the WT homodimer, deamidated-variant homodimer, and heterodimer were normalized to percentages of the total signal by using eqn (1)–(3):1
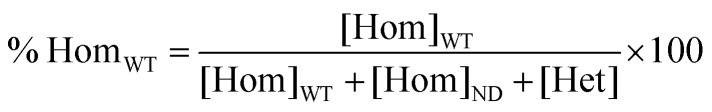
2
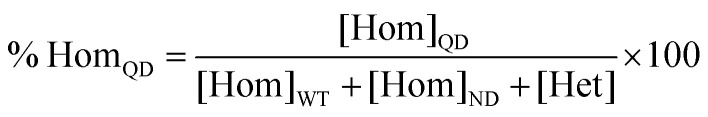
3
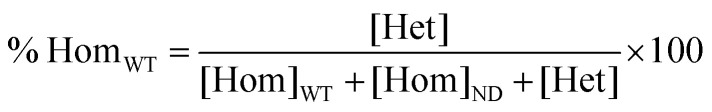
where (ND) denotes the deamidated variant. Equilibrium concentrations were substituted into eqn (4) followed by the standard Gibbs free energy of heterodimerization using eqn (5).^18^4
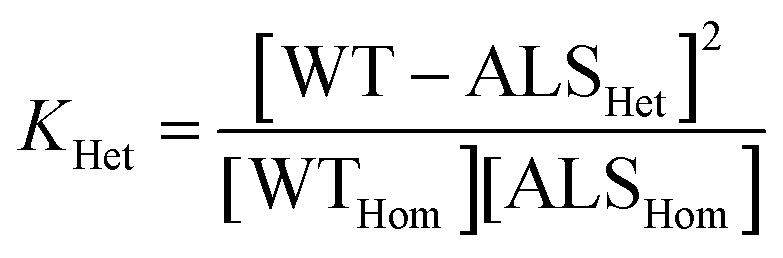

5 Δ*G*_Het_ = −*RT *ln *K*_Het_

### Kinetic analysis of heterodimer formation

Normalized heterodimer values were plotted (time *vs.* fit) in SigmaPlot by nonlinear least-squares regression to the single exponential association model eqn (6) to obtain a rate constant and calculate the *t*_1/2_eqn (7).

6 *f* = *y*_0_ + *a*(1 − e^(−*bx*)^)

7
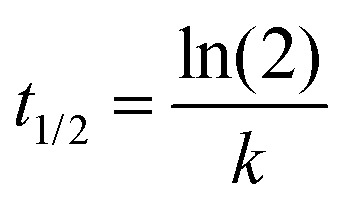


### Hydrogen/deuterium exchange

Protein samples were diluted to a final concentration of 15 µM in 99.9% D_2_O (Sigma-Aldrich) and incubated at 22 °C. 50 µL aliquots were taken at each time point (15, 30, 45, and 60 minutes), and flash frozen for mass spectrometric analysis. To quantify hydrogen back-exchange, protein samples were incubated at 90 °C for 5 minutes to generate a perdeuterated sample. For mass spectrometry analysis, each sample was thawed, resuspended in 100 µL of chilled 0.2% formic acid (pH 2.4) and immediately loaded onto a desalting column (Michrom BioResources, Inc., Auburn, CA, USA). The column was washed with 250 µL of chilled 0.2% formic acid before eluting with 80% acetonitrile, 20% of 2% formic acid. All sample preparation and experiments were executed on ice to minimize back-exchange during H/D exchange. Each measurement was completed within approximately 3 minutes of thawing using a ThermoFisher^TM^ Discovery Orbitrap mass spectrometer.

## Results & discussion

Heterodimerization of a deamidated analog of SOD1 (an Asn-to-Asp triple mutant) has been previously studied with CE. This prior study examined SOD1 at the three asparagine residues that are predicted to deamidate most rapidly in SOD1: N26, N131, and N139.^18,28^ This triple mutant, N26D/N131D/N139D SOD1, is referred to as “TD” (“triply deamidated”) SOD1. In these prior studies, we reported that the heterodimerization rate (*k*_Het_) and equilibrium free energy (Δ*G*_Het_) between WT SOD1 and TD SOD1 are comparable to those of WT SOD1 and other ALS mutants of SOD1.^18,28^ That is, the N26D/N131D/N139D substitutions did not significantly alter the rate and free energy of heterodimerization.^18,28^ It is important to note that in these prior studies and the current study, we did not prepare deamidated SOD1 by allowing the protein to react with solvent, as that would presumably require weeks to months of reaction time. Rather, we introduced Asn to Asp amino acid substitutions as this substitution is chemically identical to the reaction product of Asn deamidation to Asp (excluding iso-aspartate products).

In this study, we explore deamidated analogs of SOD1 with up to five asparagine residues converted to aspartate. We refer to the N26D/N131D/N139D/N65D/N19D SOD1 variant as the penta-variant, or PD SOD1. The five asparagine residues were selected because they are predicted to undergo deamidation most rapidly of all asparagine residues.^39^ This penta-variant serves as a model of one of the predicted states of deamidation for the SOD1 polypeptide after it exists in solution for ∼3 months (*i.e.*, ∼0.5% of SOD1 is predicted to be in this penta-deamidated form after ∼3 months).^33^ While this timeframe exceeds the lifespan of certain cell types, such timeframes do not exceed the lifetime of long-lived proteins and neural cells.^43,44^ We also constructed a quadruply deamidated analog, N26D/N131D/N139D/N19D SOD1 (QD SOD1). The first three substitutions were made in accordance with the predicted deamidation sequence, while the fourth substitution, N19, was selected due to its location in the N-terminal β-strand (dimer interface) and potential impact on heterodimerization.^38^ Both the PD and QD SOD1 variants were generated *via* site-directed mutagenesis.

### Heterodimerization was not observed between WT SOD1 and N26D/N131D/N139D/N65D/N19D (PD) SOD1

Prior to heterodimerization experiments, trypsinization and liquid chromatography-tandem mass spectrometry (LC-MS/MS) analyses were performed to confirm that WT SOD1 remained free of deamidation and that all N/D substitutions were present in each mutant. Mass spectrometry confirmed that PD SOD1 was present as a single predominant peak at 15 849 Da (Fig. S1). This observed mass aligns with the expected increase from deamidation (each deamidation increases mass by 0.984 Da), as the molecular weight of SOD1 is ∼15 844 Da per monomer (Fig. S1)^40^ Furthermore, trypsin digestion followed by LC-MS/MS indicated 100% sequence coverage and contained *X*_corr_ values above 4.4 (Fig. S2).

**Fig. 3:**
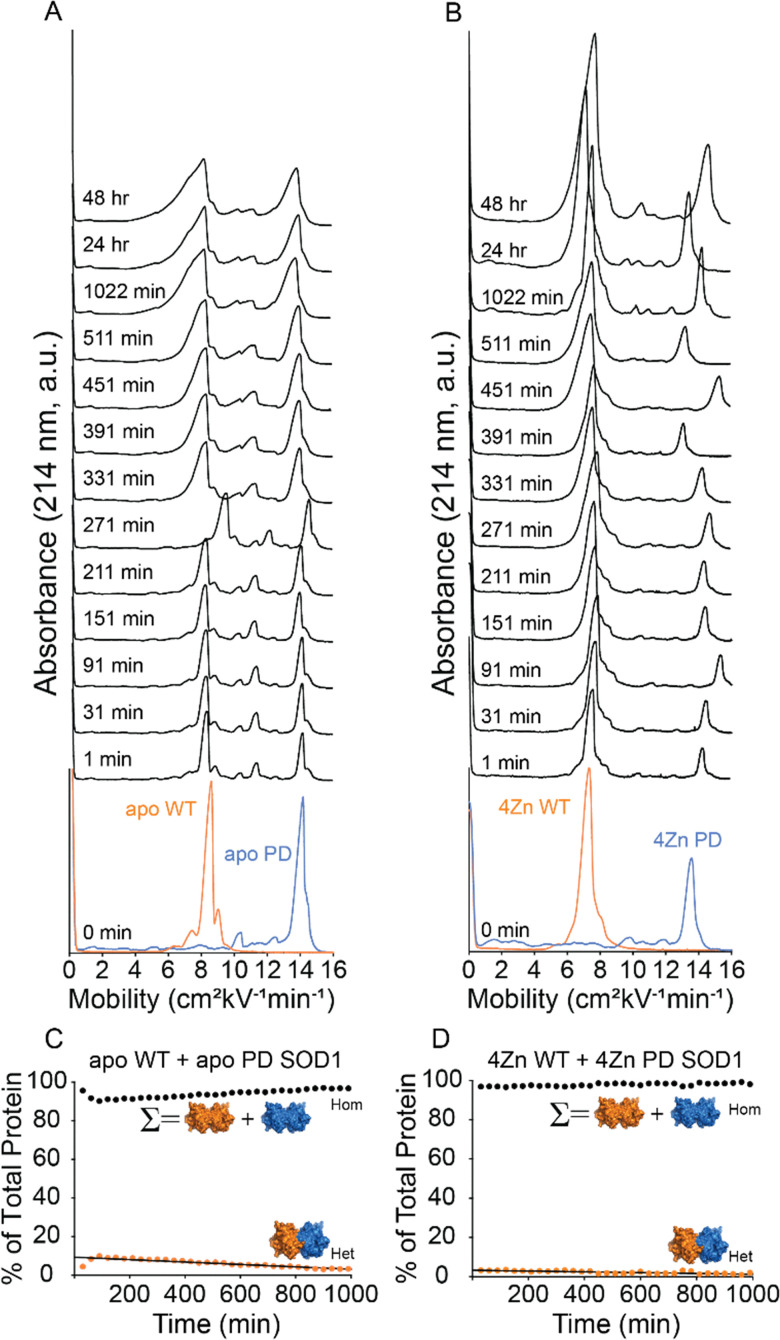
(A and B) Capillary electropherograms of WT SOD1 (orange) and PD SOD1 (blue) before and after mixing for up to 48 h. Injection times are indicated on the left of each electropherogram. Measurements were performed in triplicate. (A) WT and PD apo-SOD1, (B) Zinc-replete WT and PD SOD1. Kinetic plots (C and D) show heterodimer formation over time in both the apo and metalated states. The homodimer (Hom) value reflects the combined percent of E100K and PD homodimers, while the heterodimer (Het) is quantified separately.

Heterodimerization was initiated by mixing pure solutions of WT and mutant proteins (30 µM dimer) and monitoring heterodimerization with capillary electrophoresis (Fig. 3). Capillary electropherograms were first obtained for each unmixed homodimer (Fig. 3A and B). Capillary electrophoresis of non-isoelectric SOD1 proteins undergoing heterodimerization is characterized by a decrease in both mutant and WT SOD1 peaks (presumably, WT and mutant homodimers) and the gradual formation of a distinct “middle” peak in between the homodimer peaks (this middle peak stops increasing in intensity when equilibrium is reached).^18,27,28^ All CE experiments were performed in triplicate with peak integration of the electrophoretic signal at 214 nm conducted *via* the skim method.^18,28^

The absence of copper and zinc in the apo states of both WT and PD SOD1 were verified by inductively coupled plasma mass spectrometry (ICP-MS), confirming full demetallation of each protein. The solution of apo-WT and PD SOD1 were analyzed with CE immediately after mixing. No heterodimerization was observed over 48 hours (Fig. 3 B). A small satellite peak was present in electropherograms of PD SOD1 prior to mixing and persisted after mixing and does not represent a heterodimer.

Although the Δ*G*_Het_ could be calculated for PD and WT SOD1, *per se*, the highly positive value reflected the absence of measurable heterodimer formation. As a result, kinetic properties such as the rate constant of heterodimerization, or the “*t*_30%_” value (the time required to reach 30% heterodimer formation), and associated half-lives were not be determined (Table 1).

**Table 1:** Kinetic and thermodynamic properties of SOD1 heterodimerization for WT and mutants

	SOD1 mixture	Rate constant (*k*_Het_; 10^−2^ min^−1^)	Half-life (*t*_1/2_; min)	*k*_Het_ (*t* = min)	Δ*G*_Het_ (kJ mol^−1^)
PD + WT^a^	apoWT + apoPD	—	—	—	12.14 ± 0.16
	4ZnWT + 4ZnPD	—	—	—	16.8 ± 0.47
PD + E100K	apoE100K + apoPD	—	—	—	11.77 ± 0.83
	4ZnE100K + 4ZnPD	—	—	—	10.93 ± 0.68
QD + WT^b^	apoWT + apoQD	2.87 ± 0.35	24.26	2.18 ± 0.10	−1.91 ± 0.12
	4ZnWT + 4ZnQD	0.66 ± 0.04	105.02	5.25 ± 0.39	−4.48 ± 0.17

a PD = penta deamidated SOD1, with deamidations as follows: N26D, N131D, N139D, N65D, N19D.

b QD = quad deamidated SOD1, with deamidations as follows: N26D, N131D, N139D, N19D. Parameters of samples consisted of 15 µM homodimer ([SOD1]_total_ = 30 µM), pH 7.4, 22 °C. Each experiment was run in triplicate.

Given the absence of heterodimerization in the apo state, we next asked whether increased structural stability in the zinc-replete state might promote heterodimerization.^18,45^ Although previous studies have shown that zinc coordination by SOD1 slows its rate of heterodimerization, we wanted to ensure that the opposite effect did not exist for the apo PD-SOD1.^18^ To assess this, heterodimerization experiments were performed using zinc-replete forms of both PD and WT SOD1 (Fig. 3B). Zinc equivalents were titrated into each protein, and ICP-MS was used to confirm that the appropriate amounts of Zn were present in both homodimer proteins. After mixing of the metallated PD and WT SOD1 proteins, we observed no heterodimer peak, indicating that heterodimerization did not occur between metallated homodimers. As in the apo state, the absence of heterodimer formation precluded kinetic analysis.

### The penta-variant does not exchange subunits with E100K SOD1

**Fig. 4:**
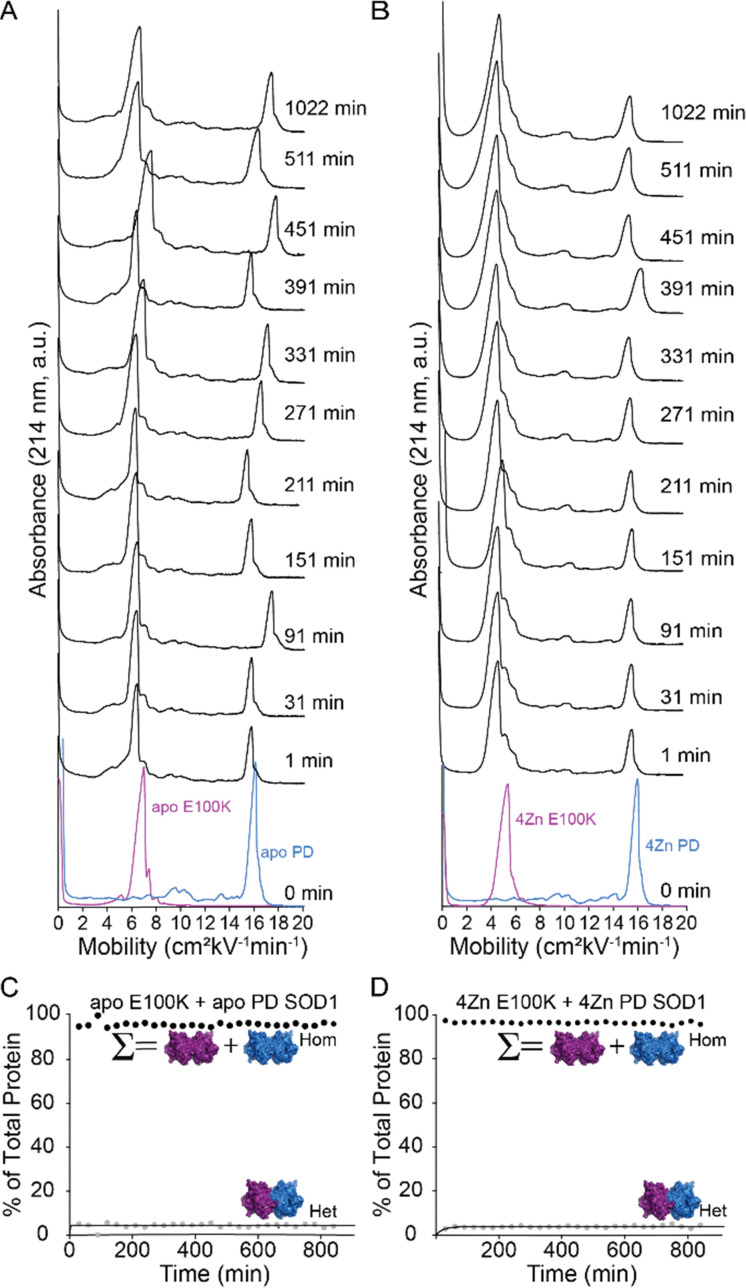
(A and B) Capillary electropherograms of E100K SOD1 (purple) and PD SOD1 (blue) before and after mixing for up to 48 h. Injection times are indicated on the left of each electropherogram. Measurements were performed in triplicate. (A) E100K and PD apo-SOD1, (B) Zinc-replete E100K and PD SOD1. Kinetic plots (C and D) show heterodimer formation over time in both the apo and metalated states. The homodimer (Hom) value reflects the combined percent of E100K and PD homodimers, while the heterodimer (Het) is quantified separately.

The PD SOD1 mutant prefers associating with itself rather than forming a dimer with the WT SOD1 subunit. The heterodimerization between PD SOD1 and the ALS-associated mutant E100K SOD1 was evaluated next. The E100K substitution decreases the formal net negative charge of the SOD1 homodimer by 4 units per homodimer (note that previous CE measurements of the net charge of E100K and WT SOD1, using protein charge ladders, suggest the change to be 3.77 ± 0.08 units at pH 7.4).^46^ The E100K SOD1 protein did not heterodimerize with the PD SOD1 protein (Fig. 4). The electropherogram showed a single peak corresponding to E100K and another to PD SOD1, with no new signal emerging upon mixing over 48 hours (Fig. 4). This suggests that PD SOD1 does not undergo subunit exchange even when paired with a more positively charged ALS-associated variant in both apo and zinc-replete states. (Fig. 4A and B).

**Fig. 5:**
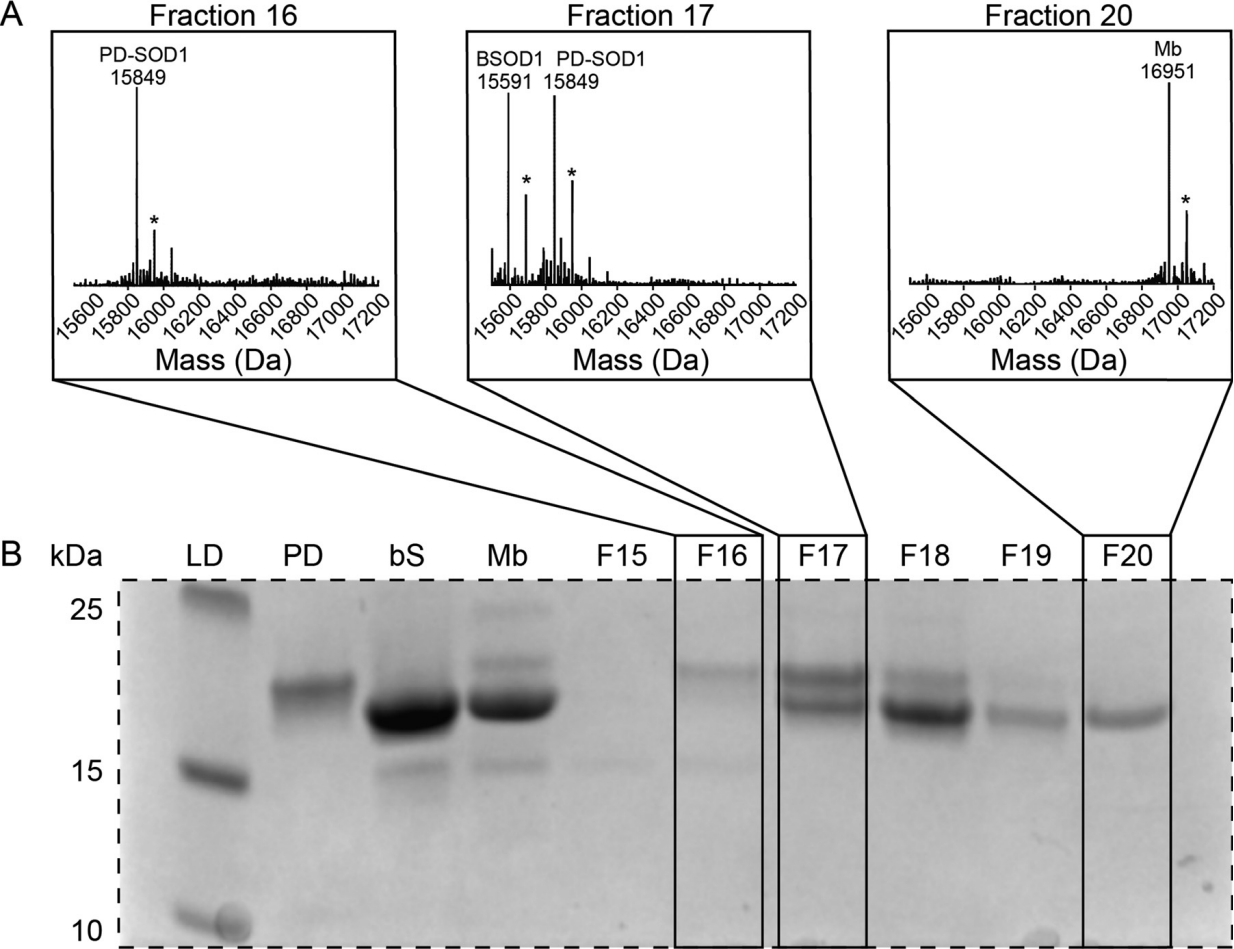
(A) Mass spectra corresponding to fractions denoted by F16, F17, and F20 (* phosphate adducts). Fraction 16 contains PD SOD1. Fraction 17 contains bovine SOD1 and PD SOD1. Fraction 20 contains myoglobin. (B) SDS-PAGE after size exclusion chromatography (SEC) using G-75 Sephadex with the ladder (LD) found in the first lane followed by standards of the proteins used in the next three lanes and fractions 15–20.

Perhaps the heterodimerization of PD-SOD1 is inhibited because the protein is not a dimer, or exists predominantly in the monomeric state. We evaluated the quaternary structure of PD SOD1 using size-exclusion chromatography (SEC) to determine whether PD SOD1 was monomeric or dimeric (Fig. 5). The molecular weight of PD SOD1 was calibrated to its elution time using internal molecular weight standards: bovine SOD1 (bSOD1) and myoglobin (Mb). These two proteins have molecular weights of 31.2 kDa (bSOD1) and 16.9 kDa (Mb). Here, if PD SOD1 elutes with Mb, it would suggest a monomeric species, whereas if PD elutes with bSOD1, it is likely dimeric. SDS-PAGE of fractions eluted from the SEC column confirmed that PD SOD1 was dimeric (Fig. 5).

The first four lanes of the gel contained the molecular weight ladder and pure protein standards, while the subsequent lanes represented SEC fractions 15 through 20. Electrospray ionization mass spectrometry (ESI-MS) of these fractions revealed that PD-SOD1 co-eluted with bSOD1, not Mb. Specifically, fractions 16 and 17 yielded mass signals of 15 849 Da and 15 591 Da, corresponding to PD and bSOD1, respectively, while a signal of 16 951 Da was detected in fraction 20, corresponding to Mb. (Fig. 5A). Note that the homodimers of bovine and human SOD1 dissociate under the denaturing conditions of ESI-MS used in these experiments. While the results of SEC suggest that PD SOD1 is a dimer, it is possible that the protein monomerizes in the electric field during CE. While it is known that both WT and ALS mutant SOD1 exist as dimers during CE (in both the metal free and fully metalated states) and do not monomerize in the electric field,^46^ there have been reports of one ALS mutant SOD1 that is a dimer in solution but monomerizes during CE (*i.e.*, A4V Zn_4_ SOD1).^47^

### Heterodimerization of the quadruple mutant SOD1 protein (N26D/N131D/N139D/N19D)

To determine if mutant analogs of quadruply deamidated SOD1 can undergo heterodimerization with WT or ALS-mutant SOD1, we constructed a quadruply deamidated (QD) variant containing the same first three deamidation sites as PD SOD1, but substituted the fourth site with N19D, located in the N-terminal β strand involved in dimer interface exchange. The molecular mass of the QD variant is ∼15 848 Da (Fig. S4).

**Fig. 6:**
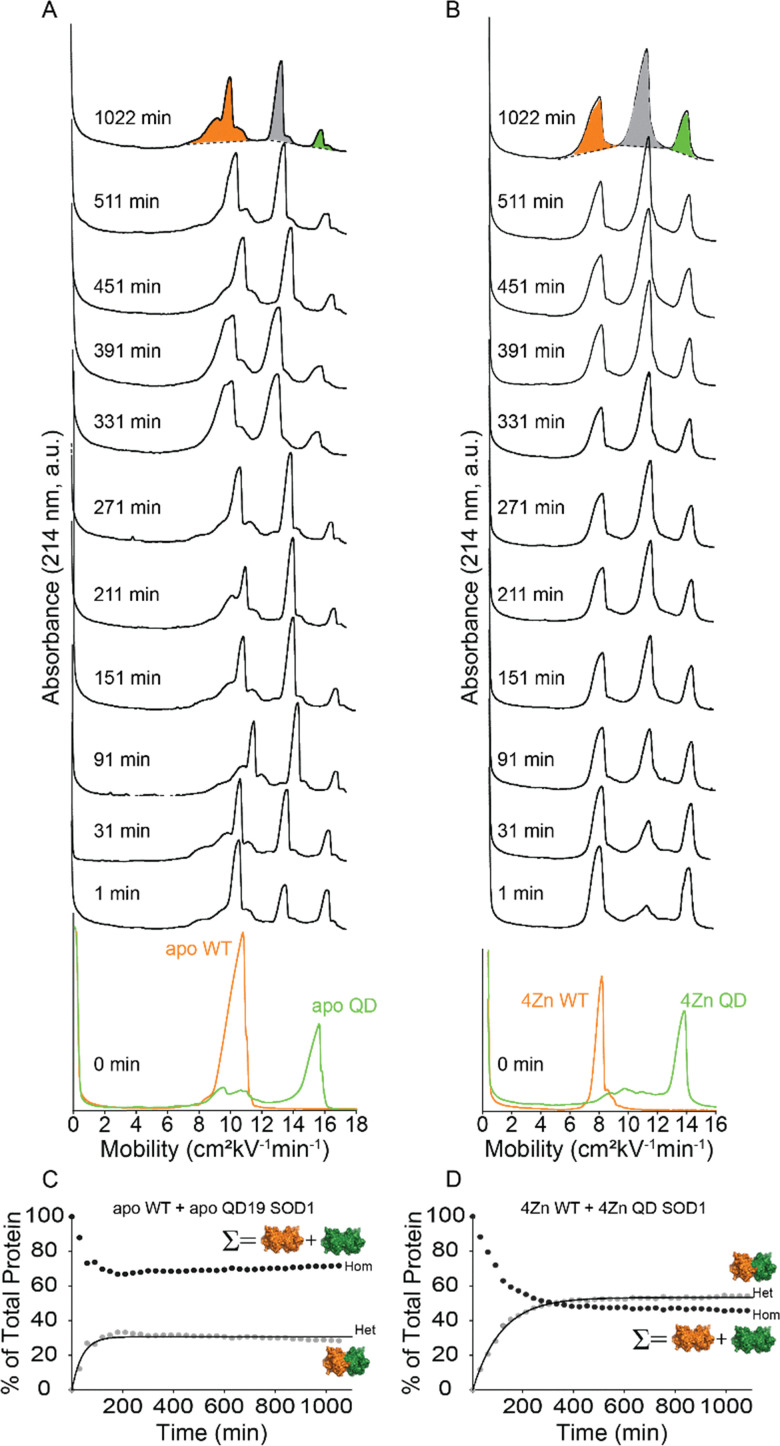
(A and B) Capillary electropherograms before and after mixing WT SOD1 (orange) and QD SOD1 (green) for up to 48 h. Injection times are indicated on the left of each electropherogram, and integration *via* the skim method is shown at 1022 minutes. Measurements were performed in triplicate. (A) WT and QD apo-SOD1, (B) Zinc-replete WT and QD SOD1. Kinetic plots (C and D) show heterodimer formation over time in both the apo and metallated states. The homodimer (Hom) value reflects the combined percent of WT and QD homodimers, while the heterodimer (Het) is quantified separately.

When this QD apo-SOD1 and WT apo-SOD1 were mixed, a heterodimer peak appeared immediately between the two homodimer peaks, indicating heterodimerization can occur between QD SOD1 and WT SOD1 proteins (Fig. 6A). Electropherograms obtained before mixing showed that QD (green) exhibited a pronounced shoulder on the left side of the peak, partially overlapping with the WT peak (orange) (Fig. 6A). As a result, the QD peak appears less uniform, giving the visual impression of a diminished peak height with an asymmetric tail that overlaps with the WT (left) and heterodimer (middle) peaks. To deconvolute and integrate this electropherogram, we applied a hybrid peak area analysis in which all three peaks were first quantified by direct integration, followed by a two-step refinement. First, the peak representing WT was individually refitted with an asymmetric peak function (using Origin Pro software) to approximate its natural shape, without contributions from the overlapping shoulder arising from the QD shoulder. Second, the area difference between this refined WT fit and the initially integrated WT peak was subtracted from WT and added to the QD peak. Although this approach yields only semi-quantitative values due to the limited resolution and fitting uncertainty, it provides consistent tracking of heterodimer formation and allows us to determine whether QD behaves differently from PD. The same correction protocol was applied to every electropherogram in the longitudinal analysis.

Plots of the relative abundance were plotted for the sum of the homodimers (Hom) and the heterodimer (Het) for each time point to determine the rate of heterodimerization for WT and QD apo-SOD1 (Fig. 6C). The exponential function eqn (6) was used to fit the plots of heterodimer or homodimer intensity *versus* time. The rate constant was calculated to be 2.87 ± 0.35 10^−2^ min^−1^. The Δ*G*_Het_ was determined to be −1.91 ± 0.12 kJ mol^−1^, with a half-life of 24.26 min (*t*_1/2_, *i.e.*, the time it takes for half of the homodimers to form heterodimers) (Table 1).

We next determined how the coordination of zinc to SOD1 affected the rate of heterodimerization of QD and WT SOD1. Heterodimerization experiments were carried out involving the Zn-replete state, where the QD and WT SOD1 were prepared with four stoichiometric equivalents of Zn^2+^ per dimer (4Zn^2+^-SOD1)/ICP-MS confirmed the coordination of 4 Zn^2+^ per dimer (Fig. 6D). All kinetic parameters of heterodimerization between WT and QD 4Zn^2+^-SOD1 are summarized in Table 1. As observed in previous studies, the heterodimerization rates for Zn^2+^-bound SOD1 were slowed by the coordination of zinc.

### Amide H/D exchange of the penta-variant

**Fig. 7:**
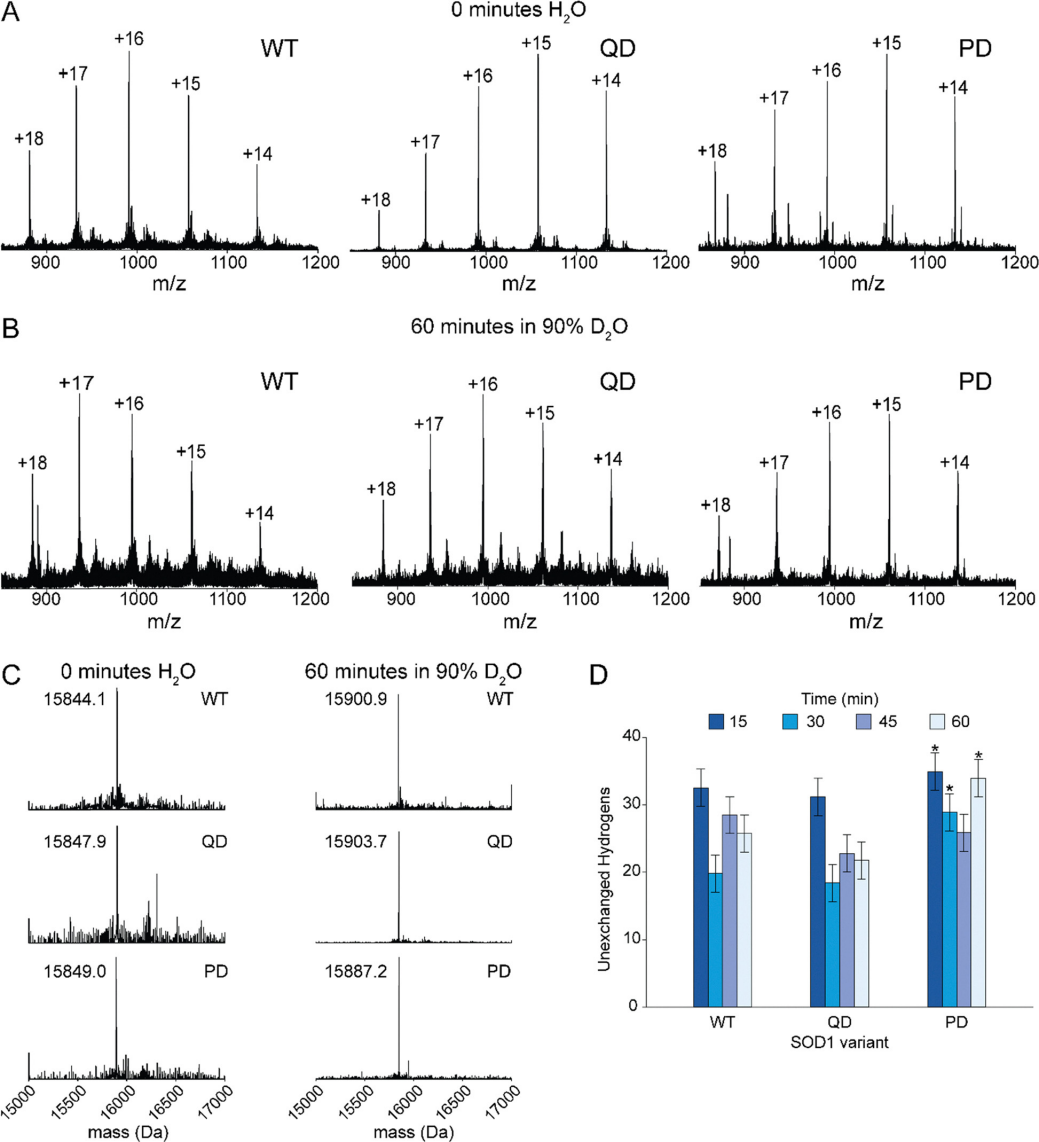
(A and B) Raw electrospray ionization mass spectra for apo WT, apo QD, and apo PD SOD1 (left to right) used to deconvolute molecular weights for 0 min (A) and 60 min in 90% D_2_O (B). (C) Deconvoluted spectra for each apo-SOD1 at 0 min (left) and 60 min (right). (D) Number of unexchanged amide hydrogens at four-time intervals (15, 30, 45, and 60 minutes) represented by the blue gradient, with error bars indicating the standard deviation from three technical replicates. Here, the number of unexchanged hydrogens reflects the amide protons that remain unexchanged after 60 minutes in 90% D_2_O, calculated as the mass difference between the native protein in D_2_O and the fully deuterated (perdeuterated) protein. Each variant was compared to WT with an unpaired *T*-test. Significance is reported as: *p ≤ 0.05.

In order to obtain information about the structural properties of PD SOD1 and QD SOD1, we analyzed PD and QD SOD1 with global hydrogen–deuterium (H/D) exchange measured by electrospray ionization mass spectrometry (Fig. 7). Previously, the rates of H/D exchange of WT and mutant SOD1 have been extensively studied with mass spectrometry, including ALS-mutant SOD1 and N/D mutants that mimic the singly, doubly, and triply deamidated forms of SOD1.^33,48,49^ Rates of exchange of WT and ALS-mutant SOD1 have also been previously examined at different post-translational states (metalation and disulfide states).^18^ The SOD1 monomer typically has between 25 and 35 unexchanged hydrogens after 60 min in 90% deuterated water.^33,50^ Some ALS mutations, such as A4V, lead to intrinsically disordered nascent proteins (disulfide-reduced, apo proteins).^47,49,50^ In the types of electrospray ionization mass spectrometry experiments used in these prior studies, and in the current study, the dimeric protein monomerizes during ionization. For this reason, the number of unexchanged hydrogens are measured per monomer.

The QD apo-SOD1 exhibited deuterium uptake nearly similar to WT at all four time points (15, 30, 45, and 60 minutes), retaining 31.2 ± 2.8, 18.4 ± 2.8, 22.7 ± 2.8, and 21.7 ± 2.8 unexchanged amide hydrogens per subunit, respectively (Table S1). For all time points, an unpaired two-tailed Welch's *T*-test was conducted, with *p*-values > 0.05 (Table S1). The PD apo-SOD1protein showed a marked increase in protection, with 34.9 ± 2.8, 28.9 ± 2.8, 25.9 ± 2.8, and 33.9 ± 2.8 unexchanged hydrogens per monomer at the same time points (Table S1). For three out of the four timepoints (15, 30, and 60 minutes), the number of unexchanged hydrogens vary significantly from the WT and PD variants (*p* ≤ 0.05, Table S1). These data align with prior reports that deamidation perturbs the electrostatic loop and alters SOD1 dynamics.^33,51^ Although the substitution of the first four asparagine have modest effects on the rate of H/D exchange, the substitution of the fifth asparagine alters the structure of 5–10 backbone amide groups (residues) in each subunit so that they do not exchange with solvent. While this decrease in rate might be due to decreases in solvent accessibility or tightened H-bonding of these amides, it is also possible that this effect is caused by changes in the electrostatic environment around the amide and not changes in H-bonding or solvent accessibility. Previous reports have shown that increases in the net negative charge of proteins (*via* lysine acetylation) can slow rates of amide H/D exchange by a similar magnitude, without altering the structure.^52,53^ These effects are attributed to increases in the transition state energy of the anionic amide intermediate that forms during base-catalyzed amide H/D exchange at neutral pH.^52,53^

### Biological significance of poly-deamidation in SOD1

Generally, the deamidation of asparagine can be a relevant solvent-mediated post-translational modification for proteins with very long half-lives (*i.e.*, extremely long-lived proteins, ELLPs);^54^ for proteins in cell types with limited turnover capacity, such as red blood cells or post-mitotic neurons; and in specific cases of proteins where deamidation functions as a chemical signal.^55^ In motor neurons, the SOD1 protein is thought to be long-lived as it undergoes slow component B (SCB) axonal transport at ∼1–2 mm day.^56^ Therefore, an SOD1 protein synthesized in the soma of a one-meter axon may require more than a year to reach the distal terminal.^56^ Thus, the types of poly-deamidated SOD1 polypeptides that we modeled in this study with successive N/D substitutions might exist (in small amounts) in motor neurons and perhaps in other cell types.^57^

The results of this study suggest that the deamidation of asparagine can affect the heteromeric state of SOD1 within motor neurons. Foremost, these results suggest that the WT SOD1 protein will cease to undergo heterodimerization when five particular asparagine residues become deamidated to aspartate. In as much as heterodimerization reactions between WT and ALS-mutant SOD1 are linked to the chemical etiology of motor neuron death in SOD1-linked ALS, it seems that asparagine deamidation of *multiple* residues might have a preventative role in ALS. However, it must be remembered that asparagine deamidation of one particular asparagine residue in SOD1 (N139) can be toxic *per se*, as at least one ALS mutation in the SOD1 gene is chemically identical to the products of asparagine deamidation (*i.e.*, N139D SOD1 is an ALS linked variant).^58,59^ Thus, the effects of asparagine deamidation might be a double-edged sword in the context of ALS.

It must be noted that the deamidation of all 7 asparagine residues have been detected in SOD1 (with mass spectrometry) from spinal cord tissue of patients who succumbed to ALS, however, only deamidation at sites N26, N53, and N131 were considered significant (excluding N139, N65, N19, and N86).^40^

In as much as heterodimerization of SOD1 is important in the maturation of the unstable nascent protein to the fully mature, thermostable protein, it is possible that deamidation could affect maturation. The potential inability of asparagine-deamidated SOD1 to heterodimerize may also impair the maturation or stabilization of newly synthesized, monomeric forms of the SOD1 protein, as illustrated in Fig. 1. This could lead to an accumulation of immature SOD1 proteins that are less stable (and more prone to aggregation) than mature SOD1 which would stress the cell's protein quality control systems.^60,61^

## Conclusion

The central observations of this study, that asparagine-to-aspartate substitutions completely abolish heterodimerization in SOD1, were a surprise to us. That is, we had no reasons to suspect that deamidation of asparagine would completely abolish heterodimerization, or even slow heterodimerization for that matter. We had assumed that N/D substitutions would accelerate heterodimerization by destabilizing the dimer interface, like other amino acid substitutions and post-translational modifications to the very stable SOD1 dimer. Consequently, we have no clear explanation for the mechanism of this effect. Nevertheless, the results clearly suggest that extensive deamidation can impair heterodimerization. Whether this loss of function reflects a purely cumulative destabilization or is the result of influence by one or two asparagine residues (*e.g.*, N65) remains unresolved and will be the subject of a separate mechanistic study. The quadruply deamidated variant (QD), with N19 substituted rather than the next sequential site N65, still underwent heterodimerization, implying that positional context may be important. Future computational and experimental studies might be able to shed light on the mechanism by which these amino acid substitutions inhibit heterodimerization.

Alas, this study raised more questions than it answered in part because the exact roles of heterodimerization in the normal and aberrant function of SOD1 are not clearly understood. Broadly, the results highlight how seemingly subtle post-translational modifications can accumulate over a protein's lifetime and remodel its conformational ensemble. In long-lived cells such as motor neurons, progressive deamidation may act as a beneficial molecular clock that puts SOD1 into an inactive state that cannot heterodimerize with newly synthesized proteins. Or, perhaps asparagine deamidation of multiple residues in SOD1 acts as an age-related modification that abolishes an important reaction of SOD1—heterodimerization—that is somehow important for the natural function of the SOD1 protein.

Regardless, the results of this study and previous studies suggest that deamidation of asparagine residues in SOD1 will likely have ambivalent effects on its function per the specific residue undergoing deamidation. Foremost, the deamidation of certain single residues, *e.g.*, N139, are equivalent to ALS-linked SOD1 mutations such as N139D. However, it is unlikely that deamidation of a single “slow” residue such as N139 will occur without deamidation of other far more rapidly converting residues such as N26. In contrast, the sequential deamidation of multiple residues might cause mutant SOD1 to form less toxic homodimers, shutting down heterodimer-mediated gain of function. This raises the intriguing possibility that deamidation at some (not all) sites might be protective in SOD1-ALS.

More broadly, in terms of the normal antioxidant function of SOD1 and protein–protein interactions involving SOD1, what are the possible effects of Asn deamidation? As far as deamidation affects dimer stability or protein–protein interactions involving SOD1 and other signaling factors, it could affect the cell's signaling pathways^62–64^ and cellular redox balance^65^ which modulates metabolism, signaling, and apoptosis.^66–69^

## Author contributions

MG: conceptualization, formal analysis, methodology, investigation, visualization, writing – original draft. TJL: investigation, formal analysis, visualization. EAA: writing – review & editing, visualization. SP: resources. NSR: resources. MTG: data curation. BFS: conceptualization, funding acquisition, methodology, resources, supervision, writing original draft. All authors contributed to writing – review & editing.

## Conflicts of interest

The authors declare no conflict of interest.

## Supplementary Material

CB-007-D5CB00225G-s001

## Data Availability

The data is provided in the manuscript and supplementary information (SI). Supplementary information is available. See DOI: https://doi.org/10.1039/d5cb00225g.
