# Supplementary material for: Does sod1 encode a molecular clock? Mutations that mimic asparagine deamidation inhibit heterodimerization with ALS-mutant SOD1
Source: RSC Chem Biol. 2026 Jan 23;7(3):473–84. doi: 10.1039/d5cb00225g (PMC12829469; doi:10.1039/d5cb00225g)
Supplement: CB-007-D5CB00225G-s001 [file CB-007-D5CB00225G-s001.pdf]

Supplementary Information (SI) for RSC Chemical Biology.
This journal is © The Royal Society of Chemistry 2026

** Supporting Information **

Does *sod1* encode a molecular clock? Mutations that mimic asparagine deamidation inhibit heterodimerization with ALS-mutant SOD1.

Mayte Gonzalez, Travis J. Lato, Emily A. Alonzo, Soeun Park, Morgan T. Green, Natalia Soto-Rodriguez, Bryan F. Shaw*

Department of Chemistry and Biochemistry, Baylor University, Waco, TX

*To whom correspondence should be addressed: bryan_shaw@baylor.edu

Key Words: Deamidation, heterodimerization, superoxide dismutase, motor neuron disease

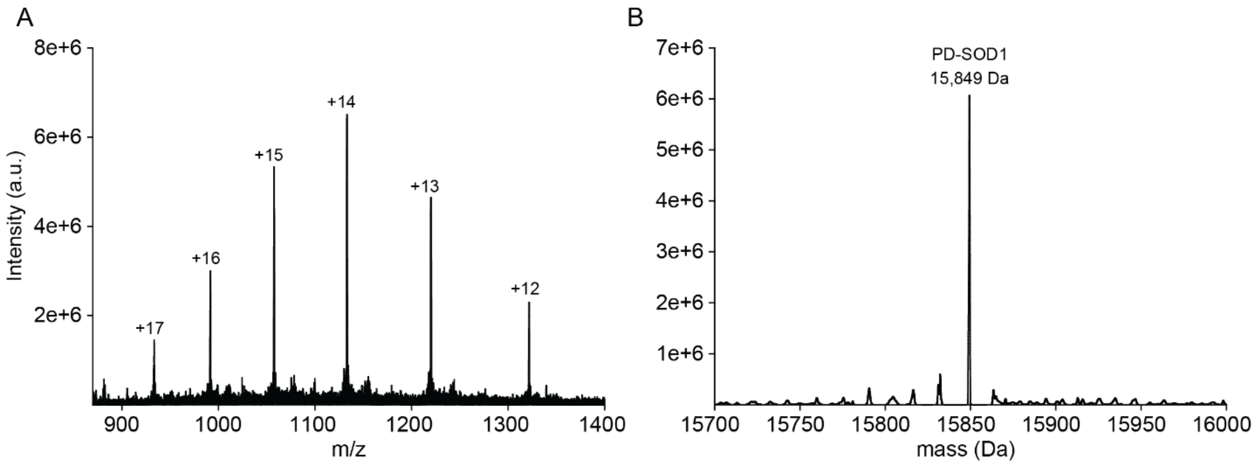

Figure S1. Mass spectra of PD-SOD1. A. Raw electrospray ionization mass spectrum showing the charge state distribution. (B) Deconvoluted spectrum displaying the molecular mass of variant (monomerization occurs during ionization).

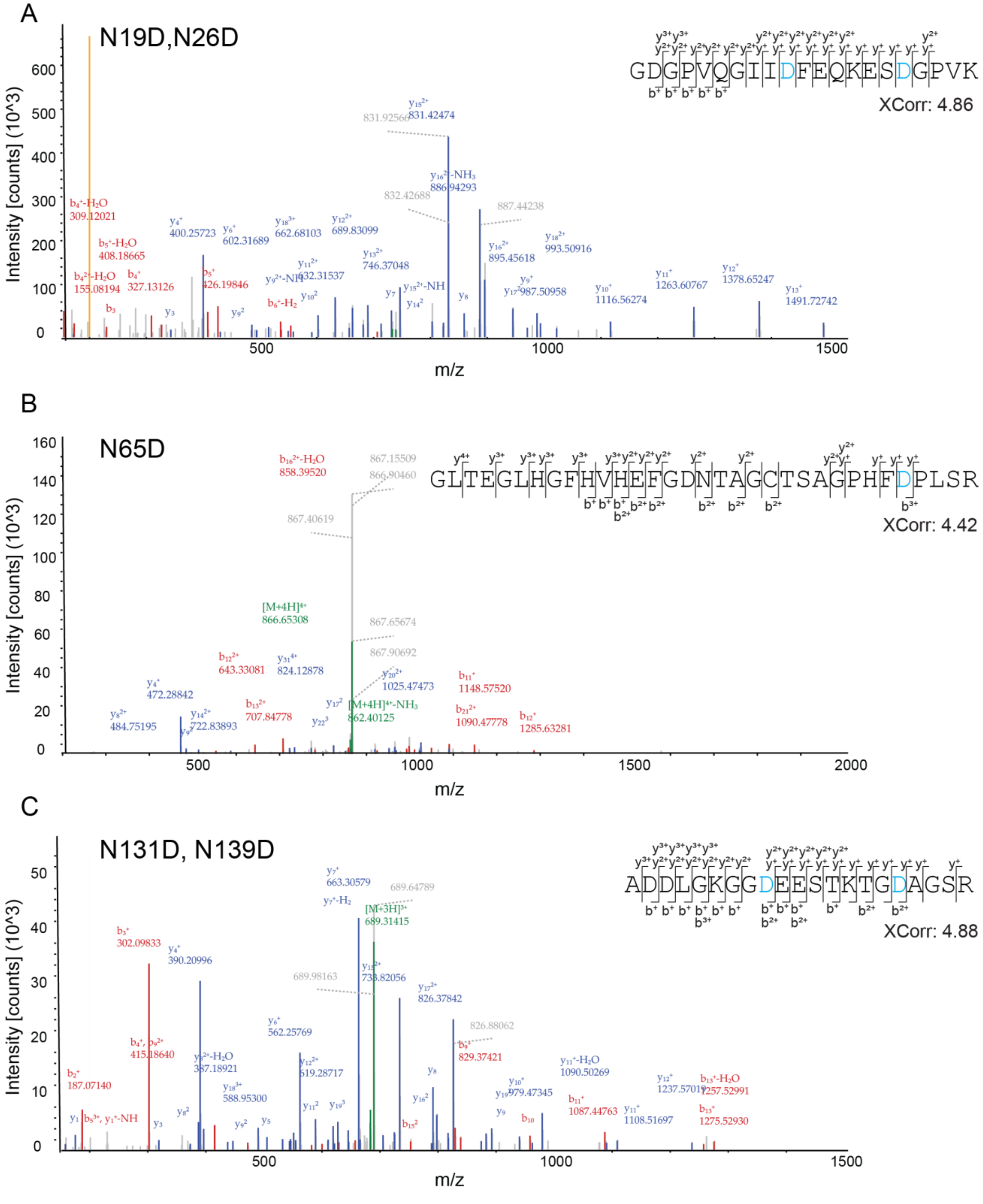

Figure S2. Representative MS/MS spectra for tryptic fragments of PD-SOD1 with corresponding peptide fragments. Shown are y and b ions and deamidated asparagine residues shown in blue. A. MS/MS spectra for the peptide fragment containing N19 and N26D. B. MS/MS spectra for the peptide fragment containing N65D. C. MS/MS spectra for the peptide fragment containing N131 and N139D.

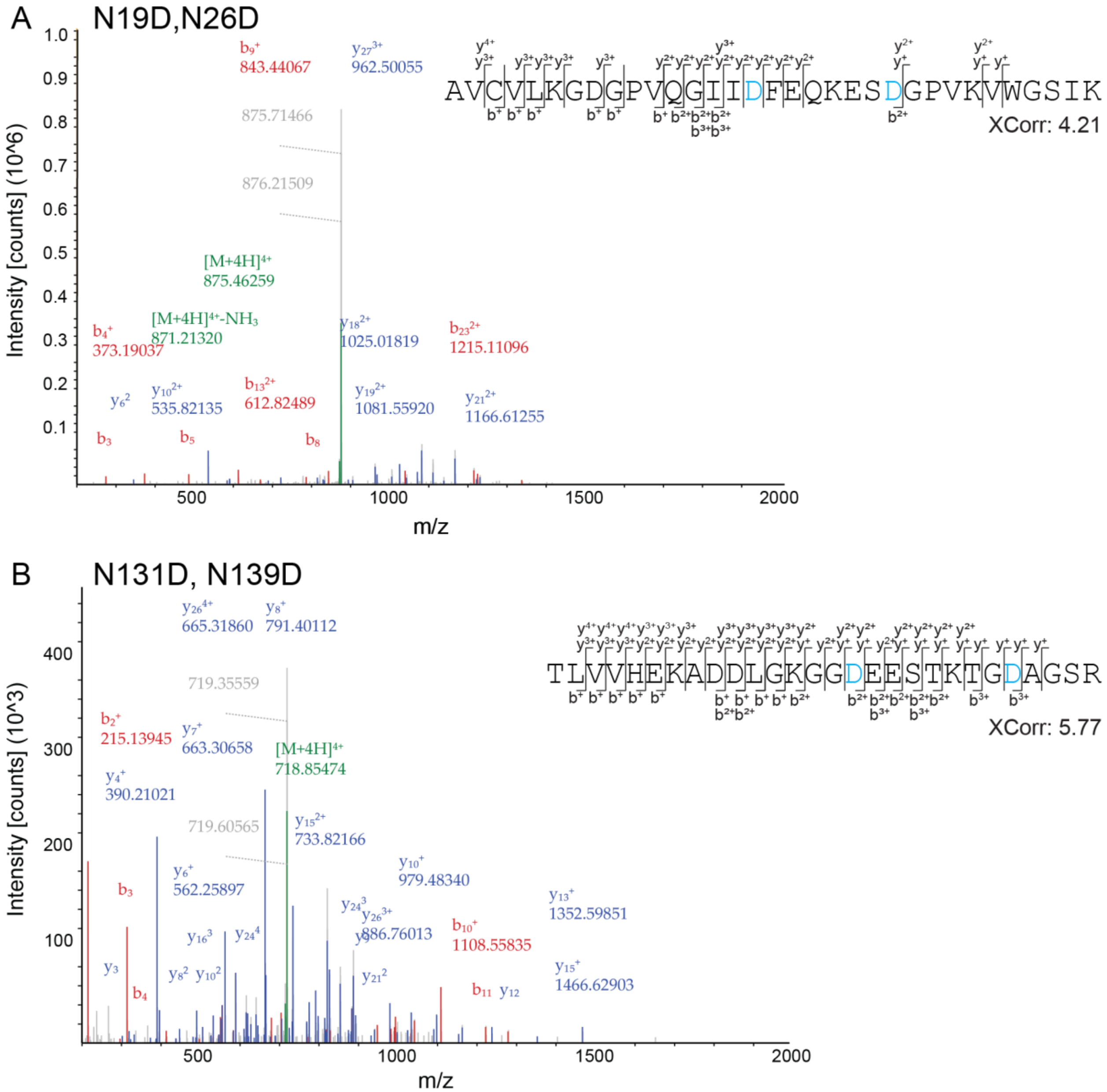

Figure S3. Representative MS/MS spectra for tryptic fragments of PD-SOD1 with corresponding peptide fragments. Shown are y and b ions and deamidated asparagine residues shown in blue. A. MS/MS spectra for the peptide fragment containing N19 and N26D. B. MS/MS spectra for the peptide fragment containing N131 and N139D.

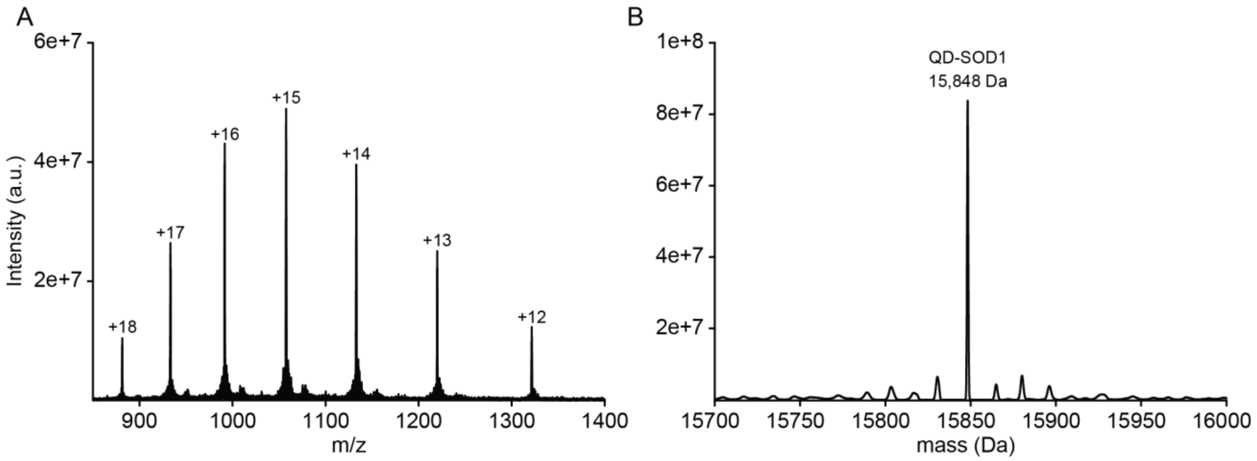

Figure S4. Mass spectra of QD-SOD1. A. Raw electrospray ionization mass spectrum showing the charge state distribution. (B) Deconvoluted spectrum displaying the molecular mass of variant (monomerization occurs during ionization).

Table S1. Number of unexchanged hydrogens for WT, QD, and PD SOD1 are presented as the mean ± std, n = 3. P-values were calculated using an unpaired Welch's T-test.

Time (mins)	Unexchanged Hydrogens WT SOD1	Unexchanged Hydrogens QD SOD1	Unexchanged Hydrogens PD SOD1	P-value WT and QD	P-value WT and PD	P-value QD and PD
15	32.5104 ± 2.7450	31.1923 ± 2.7567	34.9172 ± 2.7567	0.0599	0.0059	0.0051
30	19.7993 ± 2.7448	18.4169 ± 2.7539	28.8968 ± 2.7543	0.5447	0.0021	0.0282
45	28.4927 ± 2.7510	22.7849 ± 2.7509	25.8649 ± 2.7608	0.0556	0.2541	0.0629
60	25.7411 ± 2.7500	21.7706 ± 2.7521	33.9240 ± 2.7564	0.1344	0.0367	0.0074
